# Supplementary figures and images for: Quantification of caffeine in coffee cans using electrochemical measurements, machine learning, and boron-doped diamond electrodes
Source: PLoS One. 2024 Mar 26;19(3):e0298331. doi: 10.1371/journal.pone.0298331 (PMC10965095; doi:10.1371/journal.pone.0298331)

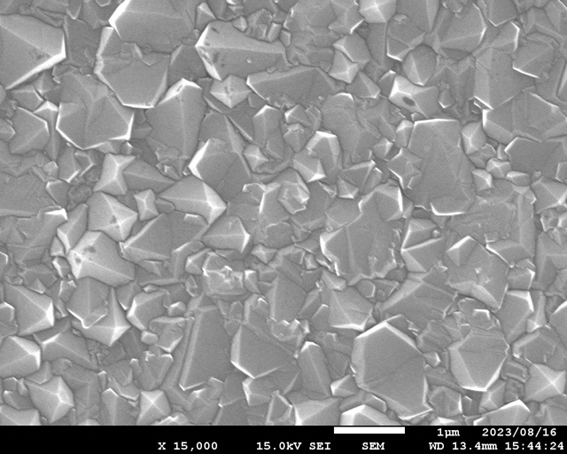

Supplement: S1 Fig — (TIF) [file pone.0298331.s001.tif]

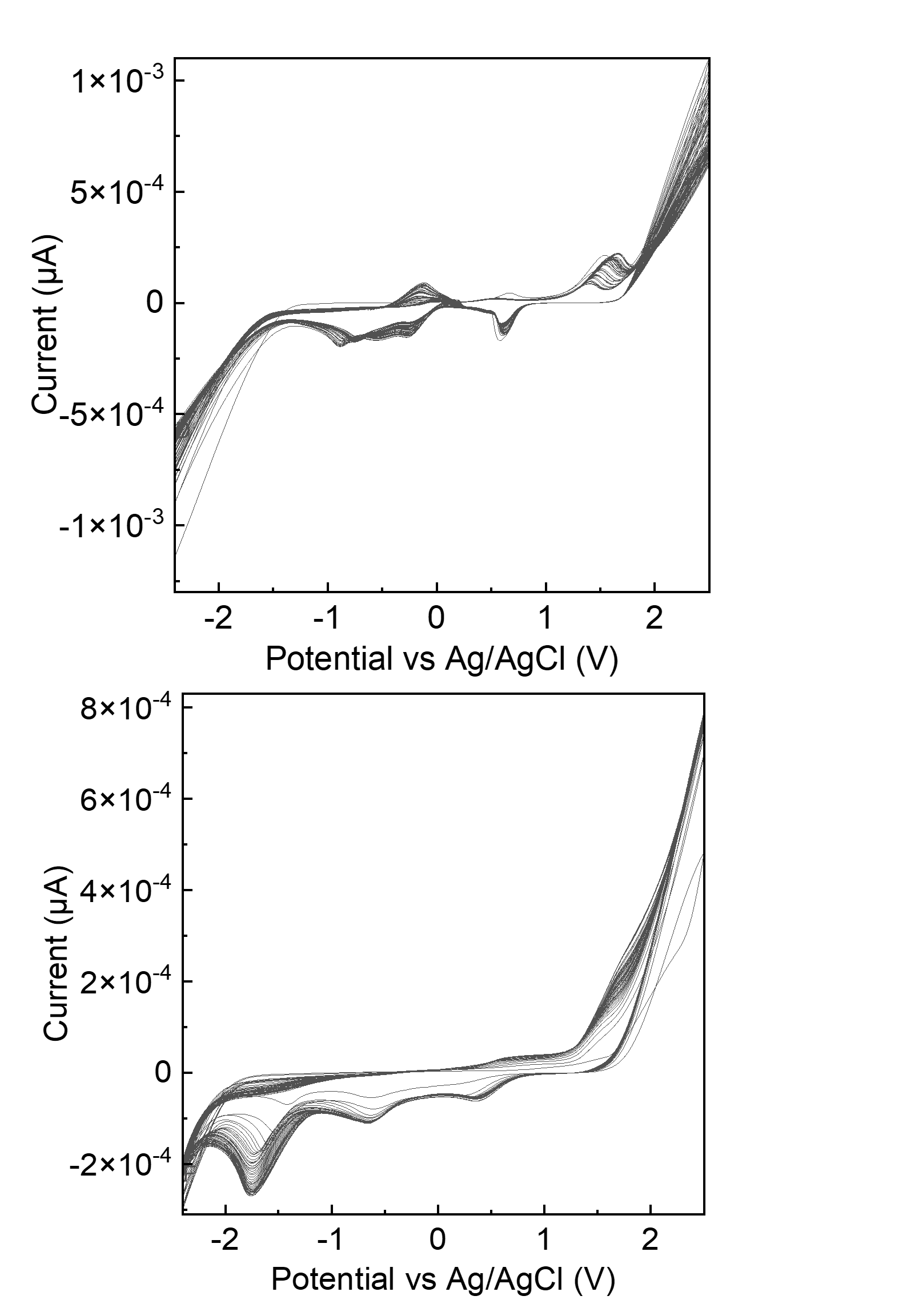

Supplement: S2 Fig — A) 100 cycles of the gold electrode in NaCl solution. B) 100 cycle of glassy carbon electrode in NaCl solution. (TIF) [file pone.0298331.s002.tif]

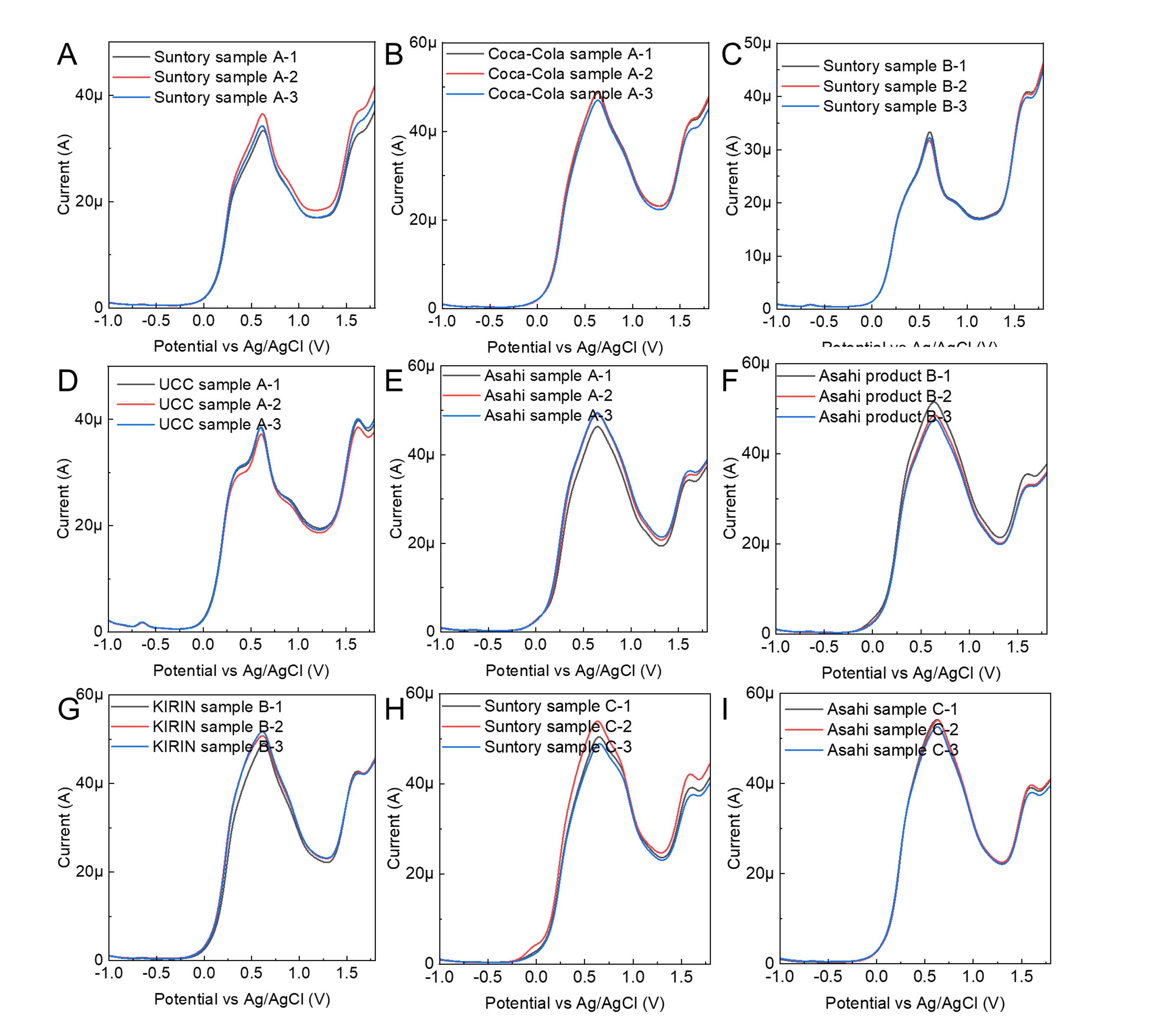

Supplement: S3 Fig — A-I) Results of 10 coffee samples from five companies measured repeatedly by SWV. (TIF) [file pone.0298331.s003.tif]
