# Supplementary material for: Quantification of caffeine in coffee cans using electrochemical measurements, machine learning, and boron-doped diamond electrodes
Source: PLoS One. 2024 Mar 26;19(3):e0298331. doi: 10.1371/journal.pone.0298331 (PMC10965095; doi:10.1371/journal.pone.0298331)
Supplement: S1 Table — (DOCX) [file pone.0298331.s004.docx]

| Name | pH | salt equivalent amount (g/L) |
| --- | --- | --- |
| Caffeine in 1 g/L NaCl | 7.45 | 1 |
| Suntory product | 6.50 | 1.1 |
| KIRIN product | 6.08 | 0.4 |
| Suntory product | 6.05 | 1 |
| Georgia product | 6.54 | 1 |
| KIRIN product | 6.54 | 0.7 |
| Asahi product | 6.42 | 0.5 |
| Suntory product | 6.67 | 1 |
