# Supplementary material for: Quantification of caffeine in coffee cans using electrochemical measurements, machine learning, and boron-doped diamond electrodes
Source: PLoS One. 2024 Mar 26;19(3):e0298331. doi: 10.1371/journal.pone.0298331 (PMC10965095; doi:10.1371/journal.pone.0298331)
Supplement: S2 Table — (DOCX) [file pone.0298331.s005.docx]

| Name | Formula | Calc. MW | Area (Max.) |
| --- | --- | --- | --- |
| Isocaffeine | C8 H10 N4 O2 | 194.08018 | 32729950461.4442 |
| D-(-)-Quinic acid | C7 H12 O6 | 192.0630 | 5,991,221,351 |
| Acesulfame | C4 H5 N O4 S | 162.9933 | 1,451,531,927 |
| Xylitol | C5 H12 O5 | 152.0679 | 1,426,348,703 |
| Oleamide | C18 H35 N O | 281.2716 | 1,191,475,937 |
| Citric acid | C6 H8 O7 | 192.0268 | 1,140,908,980 |
| D-(-)-Quinic acid | C7 H12 O6 | 192.0630 | 928,179,045 |
| Chlorogenic acid | C16 H18 O9 | 354.0956 | 574,740,604 |
| Hexadecanamide | C16 H33 N O | 255.2560 | 552,666,100 |
| D-(-)-Quinic acid | C7 H12 O6 | 192.0631 | 480,584,499 |
| Citroflex 4 | C18 H32 O7 | 360.2145 | 414,774,858 |
| α-Lactose | C12 H22 O11 | 342.1158 | 217,466,467 |
| Neochlorogenic acid | C16 H18 O9 | 354.0953 | 195,299,691 |
| 4-Methylumbelliferone | C10 H8 O3 | 176.0474 | 190,720,541 |
| D-(-)-Quinic acid | C7 H12 O6 | 192.0631 | 165,782,607 |
| D-(-)-Quinic acid | C7 H12 O6 | 192.0630 | 152,826,130 |
| Indole-3-butyric acid | C12 H13 N O2 | 203.0947 | 130,499,314 |
| Indole-3-butyric acid | C12 H13 N O2 | 203.0947 | 130,395,004 |
| L-Pyroglutamic acid | C5 H7 N O3 | 129.0427 | 127,987,166 |
| D-(-)-Quinic acid | C7 H12 O6 | 192.0632 | 109,484,322 |
| 2-[(Dimethylamino)methylidene]indan-1-one | C12 H13 N O | 187.0996 | 107,696,302 |
| trans-Zeatin | C10 H13 N5 O | 219.1121 | 103,245,274 |
| 3-(2-methylpropyl)-octahydropyrrolo[1,2-a]pyrazine-1,4-dione | C11 H18 N2 O2 | 210.1368 | 71,298,245 |
| trans-Zeatin | C10 H13 N5 O | 219.1121 | 59,943,064 |
| 4-Methylumbelliferone | C10 H8 O3 | 176.0474 | 57,519,230 |
| 3,4,5-trihydroxycyclohex-1-ene-1-carboxylic acid | C7 H10 O5 | 174.0523 | 57,158,797 |
| 1,4-Androstadiene-3,17-dione | C19 H24 O2 | 284.1776 | 55,822,567 |
| Methylsuccinic acid | C5 H8 O4 | 132.0417 | 55,530,206 |
| Metharbital | C9 H14 N2 O3 | 198.1005 | 51,181,593 |
| Acesulfame | C4 H5 N O4 S | 162.9937 | 48,262,941 |
| D-(-)-Quinic acid | C7 H12 O6 | 192.0631 | 43,958,793 |
| Terbutaline | C12 H19 N O3 | 225.1365 | 42,526,296 |
| Mesaconic acid | C5 H6 O4 | 130.0260 | 41,888,766 |
| 3,4-Dihydroxyphenylpropionic acid | C9 H10 O4 | 182.0578 | 37,522,049 |
| 1,5-Anhydro-D-glucitol | C6 H12 O5 | 164.0680 | 36,549,311 |
| 4-Methylumbelliferone | C10 H8 O3 | 176.0474 | 36,529,856 |
| 3,4,5-trihydroxycyclohex-1-ene-1-carboxylic acid | C7 H10 O5 | 174.0524 | 35,873,502 |
| 4-Acetamidobenzoic acid | C9 H9 N O3 | 179.0580 | 35,223,880 |
| Acetyl-L-carnitine | C9 H17 N O4 | 203.1159 | 30,665,939 |
| D-(-)-Quinic acid | C7 H12 O6 | 192.0632 | 28,668,353 |
| Neochlorogenic acid | C16 H18 O9 | 354.0956 | 27,908,305 |
| 2-Hydroxyphenylalanine | C9 H11 N O3 | 181.0740 | 25,460,916 |
| D-(-)-Mannitol | C6 H14 O6 | 182.0787 | 23,345,404 |
| 2,4-Diaminoanisole | C7 H10 N2 O | 138.0794 | 23,092,164 |
| D-(-)-Quinic acid | C7 H12 O6 | 192.0634 | 21,292,205 |
| NP-000587 | C16 H18 O8 | 338.1012 | 20,846,104 |
| Glucuronic acid-3,6-lactone | C6 H8 O6 | 176.0318 | 20,745,877 |
| 3,4,5-trihydroxycyclohex-1-ene-1-carboxylic acid | C7 H10 O5 | 174.0524 | 20,258,224 |
| 3-Coumaric acid | C9 H8 O3 | 164.0471 | 17,778,573 |
| trans-Aconitic acid | C6 H6 O6 | 174.0160 | 16,741,321 |
| 2,4-Diaminoanisole | C7 H10 N2 O | 138.0794 | 15,429,759 |
| 2,4,6-Trihydroxyacetophenone | C8 H8 O4 | 168.0420 | 13,788,997 |
| Caffeic acid | C9 H8 O4 | 180.0422 | 13,439,429 |
| Propylparaben | C10 H12 O3 | 180.0785 | 13,244,558 |
| 3-Coumaric acid | C9 H8 O3 | 164.0471 | 11,308,350 |
| 10-HDA | C10 H18 O3 | 186.1256 | 11,164,606 |
| Uric acid | C5 H4 N4 O3 | 168.0280 | 10,233,542 |
| Ferulic acid | C10 H10 O4 | 194.0581 | 9,348,342 |
| Tyrosol | C8 H10 O2 | 138.0675 | 8,237,145 |
| 3,4,5-trihydroxycyclohex-1-ene-1-carboxylic acid | C7 H10 O5 | 174.0528 | 8,217,548 |
| 3,4,5-trihydroxycyclohex-1-ene-1-carboxylic acid | C7 H10 O5 | 174.0524 | 7,982,361 |
| 2-Methylbenzoic acid | C8 H8 O2 | 136.0522 | 7,823,275 |
| Hippuric acid | C9 H9 N O3 | 179.0581 | 7,510,134 |
| Propylparaben | C10 H12 O3 | 180.0784 | 7,105,147 |
| NP-000615 | C14 H12 O4 | 244.0739 | 6,812,809 |
| Trinexapac | C11 H12 O5 | 224.0686 | 6,401,189 |
| Capryloylglycine | C10 H19 N O3 | 201.1365 | 6,203,304 |
| Methylhippuric acid | C10 H11 N O3 | 193.0738 | 5,581,725 |
| Propylparaben | C10 H12 O3 | 180.0784 | 5,227,582 |
| 2,4,6-Trihydroxyacetophenone | C8 H8 O4 | 168.0425 | 5,213,442 |
| Palmitic acid | C16 H32 O2 | 256.2403 | 5,143,664 |
| Porphobilinogen | C10 H14 N2 O4 | 226.0954 | 5,126,432 |
| Pantothenic acid | C9 H17 N O5 | 219.1108 | 5,078,922 |
| Genipin | C11 H14 O5 | 226.0844 | 4,674,204 |
| 2-(acetylamino)-3-(1H-indol-3-yl)propanoic acid | C13 H14 N2 O3 | 246.1008 | 3,896,798 |
| 3,4,5-trihydroxycyclohex-1-ene-1-carboxylic acid | C7 H10 O5 | 174.0524 | 3,888,426 |
| 2-Amino-3-(4-hydroxy-3-methoxyphenyl)propanoic acid | C10 H13 N O4 | 211.0844 | 3,796,242 |
| Gabapentin | C9 H17 N O2 | 171.1260 | 263,129 |
