# Supplementary material for: Quantification of caffeine in coffee cans using electrochemical measurements, machine learning, and boron-doped diamond electrodes
Source: PLoS One. 2024 Mar 26;19(3):e0298331. doi: 10.1371/journal.pone.0298331 (PMC10965095; doi:10.1371/journal.pone.0298331)
Supplement: Attachment — Submitted filename: plos one comments.docx [file pone.0298331.s006.docx]

The present work report **“Quantification of Caffeine in Coffee Cans Using Electrochemical Measurements, Machine Learning, and Boron-doped Diamond Electrodes”.**

In my opinion, the application of machine learning tool for current work provides an additional data in addition to the experimental results. This application is a very interesting part for the present work that provide significance to the current work.

However, there are some major concerns that must be addressed by the authors in the electrochemical experiment parts.

1. In the method section, authors have mentioned the **film thickness and boron concentration, 99 as measured by secondary ion mass spectrometry, were 5 m and >1020 cm-3**, respectively. Authors have to provide this data as no supporting data is provide for these values.
2. Authors should explain why they have selected only NaCl (1g/L) solution for the measurement during the experiment with proper explanation.
3. Authors have performed the SWV for a voltage from **-2.4 V to 2.5 V** while for Principal component analysis the selected range is **1.55 V and 1.65 V.** What is the explanation for this voltage range variation. Is there any specific reason for this?
4. In **Machine learning for quantification of caffeine in beverages** section, the values of Caffeine content given by manufacturer and the data provided after machine learning parameters has large variation. I wanted to know what is the permissible values or standard deviation that can be accepted for the comparison of experimental results and AI results in such cases.
