# Supplementary material for: Quantification of caffeine in coffee cans using electrochemical measurements, machine learning, and boron-doped diamond electrodes
Source: PLoS One. 2024 Mar 26;19(3):e0298331. doi: 10.1371/journal.pone.0298331 (PMC10965095; doi:10.1371/journal.pone.0298331)
Supplement: Attachment — Submitted filename: Reviewer comments R2.pdf [file pone.0298331.s008.pdf]

Reviewer #2: The authors answered partially the questions, and a complete response is required. All these comments should be inserted in the revised manuscript, because these comments are important to the readers. Some few were inserted in the manuscript, but all suggestions need to be used. The introduction with suggested references and others also is needed. Thus, a minor revision is required. New review is marked as "Review-R2".

Response: We thank you for your careful reviewing of our manuscript. We have revised the MS to comply with your comments. Responses to each comment can be found below. Please check it out.

Review-R2: A Table for comparison of data or features, highlighting is possible being optimized or not. Thus, please insert it in the revised manuscript.

Response: Comparisons with other studies were conducted. The comparison focused specifically on the number of pretreatments and time. Revisions were made as follows. Please check the revised sections.

PAGE 21, LINE 308-328

The principle, number of pretreatments to perform the measurement with high accuracy, and expected measurement time, including pretreatment, were compared with other studies (Table 5). Compared to standard HPLC, the number of pretreatments and measurement times were significantly higher. HPLC showed 99.2% quantitative accuracy for commercial beverages, indicating that HPLC is a simple measurement method, but its accuracy is inferior. The colorimetric and Raman spectrophotometric detection methods used a smaller number of pretreatments and measurement times to quantify commercial beverages or prepared caffeine. In both methods, a calibration curve was prepared to determine the quantity of caffeine, and it was necessary to prepare a standard for each case. The major advantage of this study is that it can be used for product evaluation without the need to create a calibration curve if it has been studied in advance. Comparisons were made with other electrochemical caffeine sensors. By fabricating an electrode with a structure suitable for caffeine and measuring under optimal solvent conditions, we succeeded in achieving ultrahigh sensitivity and 99.9% accuracy in a single step. Electrochemical sensors can be constructed with high sensitivity under optimal conditions. In this study, no pretreatment was performed, and the electrochemical

double layer formed at the electrode interface in the solution was not stable because no electrolyte was added. This leads to measurement instability and a reduced sensitivity. By utilizing machine learning, we succeeded in quantification with an accuracy of over 90% even from products containing emulsifiers without using any electrolytes, whereas it was originally necessary to establish optimal conditions as in previous studies. However, this is difficult to achieve using other electrochemical sensors.

Review-R2: The potential interferent compounds are a problem, thus, the electroactive compound with oxidation potential leads to an accurate data. Thus, it is important to comment on it. The only comment in the conclusion is not sufficient, because the article and its discussions need to be inserted in the revised manuscript.

Response: Comparisons with other studies were conducted. The comparison focused specifically on the number of pretreatments and time. Revisions were made as follows. Please check the revised sections.

Review-R2: A comparison table is possible to be constructed and to show the main highlights of the manuscript compared to others, to measure caffeine for coffee and other samples to evaluate the performance of the electrochemical methods and the method proposed here. For quantitative purposes, the limit of detection and quantification, linear range are always supplied, thus, the authors should be presented with a comparison of the method reported in the literature and the proposed method.

Response: We prepared comparison table as Table 5. Please confirm it in revised MS.

Review-R2: Ok, but a statistical test should be performed. Please insert the data and prove this possible inconsistency.

Response: We believe that you are right to your point. However, in this method, one type of coffee can be independent of one population. We believe that the t-tests for each population will differ depending on the detailed differences in the substances contained in coffee cans. We also assumed that we could randomly select one type of coffee and conduct the test, but we thought that it would not be possible to make a significant discussion because of the large population size. The validity of this study is that once we have teacher data, we can immediately quantify it. Statistical analyses will be performed

for all populations in the future.

Review-R2: Why were the recovery tests always lower than 100%?

Response: The values predicted in this study are listed in Table 2. The values in Table 2 are the absolute percentages based on the recovery rate. In Table 2, "x divided by 100 and the remainder subtracted from 100 (%)," where x refers to Recovery (%). These were used to evaluate overall coffee consumption. It was necessary to correct this and change x to the recovery (%). The mean and median values in Table 4 are obtained from the results in the first right-hand column of Table 2. Therefore, it did not exceed 100%.

Review-R2: This comment needs to be inserted in the manuscript, because 0.4-0.8 V is not used for Caffeine quantification, only the oxidation peak at 1.6 V. This high potential is easily found for other compounds; this information needs to be discussed in the revised manuscript.

Response: Thank you for pointing this out. I have added it to the revised MS. Please check it.

PAGE 12, LINE 215-218

A peak attributable to the oxidation of caffeine is also observed at 0.4-0.8 V. In this potential range, many substances, such as OH groups in organic compounds, show oxidative responses. Therefore, it is difficult to differentiate the oxidation response from foreign signals in actual commercial coffee beverages. Only the 1.6 V peak is used for actual quantification.

Reviewer #3: Authors duly revised the manuscript according to the comments. Hence, the manuscript can be acceptable in the present form. Thank you.

Response: We thank you for reviewing our manuscript and considering it suitable for publication.

Reviewer #4: All concerns have been carefully addressed and the manuscript is suitable for publication. The authors have extend the discussion section and give all parameters used in the experimental section in detail. Several figures have been improved. Other references have been added even when other reported already published have been not included for example, Martinez-Huitle group.

Response: We thank you for reviewing our manuscript and considering it suitable for publication.
